# The Effects of Intermittent Fasting Combined with Resistance Training on Lean Body Mass: A Systematic Review of Human Studies

**DOI:** 10.3390/nu12082349

**Published:** 2020-08-06

**Authors:** Stephen Keenan, Matthew B. Cooke, Regina Belski

**Affiliations:** School of Health Sciences, Swinburne University of Technology, Hawthorn, VIC 3122, Australia; mbcooke@swin.edu.au (M.B.C.); rbelski@swin.edu.au (R.B.)

**Keywords:** intermittent fasting, exercise, resistance training, body composition, lean body mass

## Abstract

Diets utilising intermittent fasting (IF) as a strategic method to manipulate body composition have recently grown in popularity, however, dietary practices involving fasting have also been followed for centuries for religious reasons (i.e., Ramadan). Regardless of the reasons for engaging in IF, the impacts on lean body mass (LBM) may be detrimental. Previous research has demonstrated that resistance training promotes LBM accrual, however, whether this still occurs during IF is unclear. Therefore, the objective of this review is to systematically analyse human studies investigating the effects of variations of IF combined with resistance training on changes in LBM in previously sedentary or trained (non-elite) individuals. Changes in body weight and fat mass, and protocol adherence were assessed as a secondary objective. This review followed the preferred reporting items for systematic reviews and meta-analyses (PRISMA) guidelines. MEDLINE, CINAHL, PubMed and SportDiscus databases were searched for articles investigating IF, combined with resistance training that reported measures of body composition. Eight studies met the eligibility criteria. LBM was generally maintained, while one study reported a significant increase in LBM. Body fat mass or percentage was significantly reduced in five of eight studies. Results suggest that IF paired with resistance training generally maintains LBM, and can also promote fat loss. Future research should examine longer-term effects of various forms of IF combined with resistance training compared to traditional forms of energy restriction. Prospero registration CRD42018103867.

## 1. Introduction

Non-linear dieting approaches, such as intermittent fasting (IF), have recently gained popularity as a method of manipulating body composition. Variations of IF include total alternate day fasting (ADF), modified ADF, time-restricted feeding (TRF) and diet breaks, amongst others [1]. Whilst research into how they affect body composition is relatively recent, fasting for religious reasons has been practiced for centuries. Each year, millions of Muslims around the world participate in the holy month of Ramadan, abstaining from food and fluid from sunrise to sunset [2]. Regardless of the reason or method of fasting, all involve extended periods of time with little or no nutritional intake. While there are many purported health benefits with fasting, including those linked to elevated body mass index and/or chronic inflammation [3,4,5,6], a potentially unfavourable consequence of energy restriction is loss of lean body mass (LBM). The maintenance of skeletal muscle mass (which is a large component of LBM), is determined by changes in rates of skeletal muscle protein synthesis (MPS) and skeletal muscle protein breakdown (MPB) [7]. Both MPS and MPB rates are influenced by energy balance. During energy balance, acute periods of positive and negative protein balance as a result of a fed and fasted state, respectively, have little impact on muscle mass as the net protein balance is neutral [8]. However, during energy restriction or fasting, rates of MPB might be accelerated and rates of MPS reduced [9,10]. Subsequently, this can result in a decline in net protein balance and may contribute, in part, to reductions in LBM.

Preservation or growth of LBM is important for a number of reasons. Firstly, given that LBM is a major determinant of basal metabolic rate (BMR), losses in LBM could have an important impact on total energy expenditure. For each kilogram of LBM lost, an estimated reduction in BMR by about 13 kcal per day has been reported [11], though estimates vary from 3–4 kcal, up to 33 kcal per kilogram [12,13]. While the amount of reduction in BMR seems trivial, especially at the lower ends of these estimates, the continued loss of LBM and reductions in BMR could subsequently compromise the efforts of individuals seeking to lose weight, and conversely, may increase the risk of weight gain. In addition, given LBM is a highly metabolically active tissue, reduced LBM may compromise aspects of metabolic health and increase the risk of disease development (i.e., type 2 diabetes) in overweight and obese individuals and/or may contribute to the development of sarcopenia and bone loss, especially in older adults [14,15]. Lastly, while not uniformly correlated [16], losses of LBM may compromise strength and physical function, with some studies reporting associated decreases in handgrip and knee-extensor strength [17]. For these reasons, strategies that prevent the loss of LBM as a result of diets incorporating fasting should be considered.

One such strategy that can attenuate LBM losses and could even result in gains in LBM during energy restriction is performing physical activity, specifically resistance training [18,19]. While the majority of research has focused on continuous energy restriction (CER), a number of emerging studies have investigated the impact of other forms of energy restriction, such as IF, combined with resistance training on body composition, including changes in LBM. However, to date, these studies have not been systematically reviewed. Therefore, the purpose of this systematic review is to assess the combined effects of IF (including Ramadan fasting) with resistance training on changes in LBM in sedentary and active (non-elite), overweight and normal weight individuals. The secondary objective is to assess the effects of the same diet intervention protocols on body fat, weight and adherence rates. This review was conducted and reported according to the preferred reporting items for systematic reviews and meta-analyses (PRISMA) guidelines [20].

## 2. Materials and Methods

This study was conducted and reported according to the PRISMA guidelines [20]. The PRISMA checklist can be found in supplementary material Table S1. A protocol was designed a priori and registered with PROSPERO, registration number = PROSPERO 2018 CRD42018103867 [21].

### 2.1. Data Sources

A systematic search was conducted using the databases MEDLINE, CINAHL, PubMed and SportDiscus for original research articles published in peer reviewed journals that had investigated the impact of IF combined with resistance training on body composition. Key search terms were: alternate day fast* OR alternat* calori* diet* OR alternate day diet* OR alternate day modified fast* OR intermittent fast* OR intermittent energy fast* OR intermittent energy restrict* OR intermittent calori* restrict* OR ADF OR time restricted feed* OR TRF OR Ramadan OR Ramadan fast* AND exercis* OR resistance exercis* OR training AND body composition. An example of a full electronic search strategy in MEDLINE can be found in supplementary material Table S2.

### 2.2. Study Selection

No restrictions were placed on publication date, as research in this area is relatively recent and limited in amount. To be included in this review, studies were required to meet the following criteria: (i) original article; (ii) interventions in humans; (iii) include a description of the fasting protocol; (iv) include a description of the exercise undertaken by participants during the trial period, and include a resistance training component; (v) include a measure of body composition and report on either body fat (as a percentage or as total fat mass) or lean body mass. Exclusion criteria included: (i) non-human studies; (ii) reviews, case studies, letters, surveys, abstracts, conference papers or duplicate reports; (iii) non-English publication; (iv) grey literature; (v) studies in elite or professional athletes. Papers were not excluded for lack of control group or comparison group, as long as they met the above inclusion criteria. Final searches were performed on 16 July 2018 and updated on 17 April 2020.

### 2.3. Data Extraction

**Figure 1:**
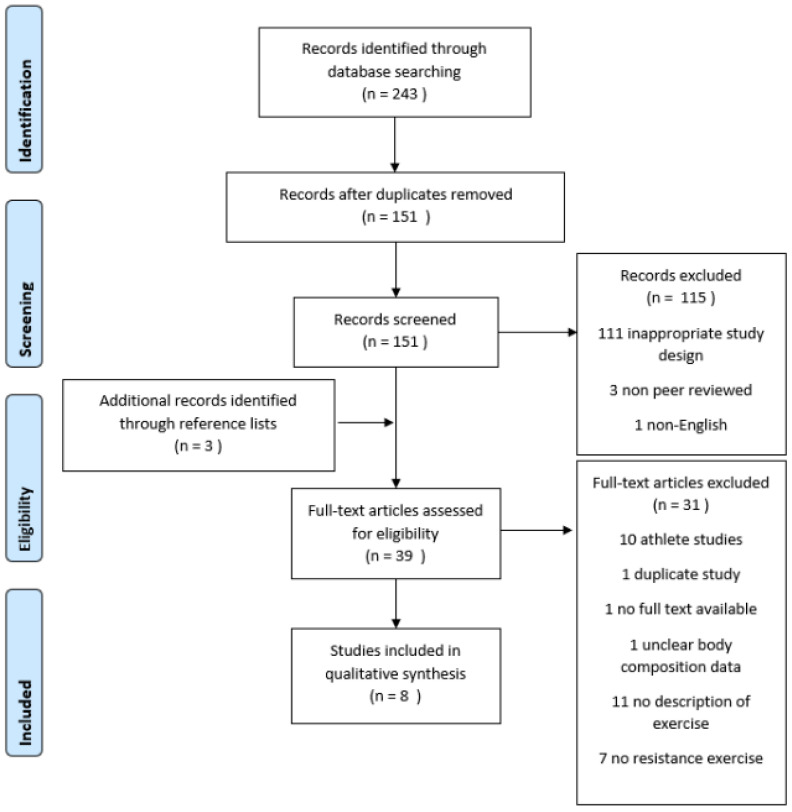
Preferred reporting items for systematic reviews and meta-analyses (PRISMA) flow diagram of study selection.

The initial database search resulted in 243 studies being returned. Of these, 36 met the inclusion criteria and underwent full-text review. The reference lists of these studies were searched for further appropriate studies, with 3 more being identified for full review. Out of these 39 studies, 8 met the inclusion criteria. From these studies, publication data, participant details, duration of study, description of fasting method and exercise protocol, mean weight and anthropometric changes, methods of anthropometric assessment and adherence rates were extracted into a custom template. The PRISMA flow diagram [20] for this search process, including reasons for study exclusion, is presented in Figure 1.

### 2.4. Quality Appraisal

The Downs and Black quality check list [22] was used to assess the risk of bias in each study consistent with previously published reviews in this field [23]. This checklist comprises 27 questions addressing reporting quality and external and internal validity. Question 14, regarding blinding of subjects, was removed as this is not seen as possible in these dietary interventions. Each question is rated as either a yes (1 point), no (0 points) or unable to determine (0 points), with 1 question rated on a 3 point scale (yes = 2 points, partial = 1 points, no = 0 points). Given varying primary outcomes of the studies included, and the difficulty of assigning a clinically meaningful difference for these outcomes, the final question relating to power analyses has been altered. Studies were awarded 1 point if they included sample size calculations, and no points if this was omitted. Maximum scores of 27 and 26 were available for randomised and non-randomised studies, respectively.

## 3. Results

Participant and study characteristics are presented in Table 1 [24,25,26,27,28,29,30,31].These include: body mass index (BMI), age, sex, number of drop outs, length of intervention period, description of fasting and exercise protocol, weight and body composition changes (effects of the intervention), method of anthropometric assessment and study quality score. Additionally, information on adherence to dietary protocols was also included in the body of this review from studies that reported it.

### 3.1. Intervention Period

Intervention periods ranged from 4 to 8 weeks. Three studies that investigated Ramadan fasting were approximately 4 weeks in duration [29,30,31]. Three studies that investigated TRF were 8 weeks in duration [24,25,26], while a fourth was 4 weeks in duration [27]. The one study that investigated modified ADF was 8 weeks in duration [28].

### 3.2. Participant Characteristics

Of the eight studies included, there were a combined total of 219 participants across all groups, including 153 males and 66 females. Twenty-seven individuals withdrew from their respective studies [25,26,27,28], leaving 192 individuals completing the interventions. A total of 120 individuals completed the IF intervention arm and 72 completed the comparison arms. The sex distribution of final numbers of completed participants could not be accurately determined, given that one study did not report the sex of withdrawn participants [28]. Eight participants from two studies were excluded from analysis due to non-compliance [25,27]. Previous exercise/training history was varied between studies, with some participants classified as recreationally active [25,31] or resistance trained [26,27,29,30]. One study provided insufficient details regarding previous exercise/training history, though it was noted participants were unfamiliar with resistance exercise [25]. The age of the participants ranged from young adults (22.0 ± 2.4 year) [25] to middle-aged adults (40.6 ± 10.0 year) [28]. BMI ranged from normal weight (22.5 ± no SD) [26] to moderately overweight (28.3 ± 4.1) [28]. Only three studies reported the BMI of participants [28,29,30]; the BMI of the remaining studies was calculated using mean baseline height and weight values. Baseline body fat percentage ranged from 12.9 ± 3.5% [31] to 34.9 ± 4.6% [28], though it should be noted that various assessment techniques were used.

### 3.3. Intervention—Fasting Protocol

Of the eight studies reviewed, three studies investigated Ramadan fasting [29,30,31], with fasting durations approximately 14–15 h per day. Only one of these studies included a control diet group [30], while the remaining Ramadan studies reported no control diet group. Four studies utilised TRF [24,25,26,27]. Of those, one study instructed participants to consume their daily intake within a 4 h window between 4 p.m. and midnight, 4 days per week, with no restrictions on food choices or overall intake [25]. On the other 3 days per week, participants were allowed ad libitum intake. Three studies instructed participants to consume all of their daily intake within an 8 h window [24,26,27]. The first of these involved participants consuming 100% of their estimated energy requirements over three regular intervals at 1 p.m., 4 p.m. and 8 p.m. every day [24]. The second study allowed intake between 12 p.m. and 8 p.m. without set intervals, aimed for participants to be in a small energy deficit (~250 kcal), and to consume a high protein diet (≥1.4 g of protein per kilogram of bodyweight per day) [26]. This study also included another TRF group with the same guidelines, but which consumed an additional 3 g of β-hydroxy β-methylbutyrate (HMB) per day. The third study allowed participants the choice of consuming their energy intake between 12 p.m. and 8 p.m., or 1 p.m. and 9 p.m. [27]. Participants aimed to restrict energy by approximately 25% of estimated requirements, and also consume 1.8 g of protein per kilogram of bodyweight per day. Comparison diet groups included: usual dietary intake with no time restrictions or scheduled times of consumption [25]; usual dietary intake, but consumed within a 13 h period and scheduled times of consumption (8 a.m., 1 p.m. and 8 p.m.) [24]; eating at self-selected intervals but with the same 250 kcal energy deficit and a protein intake of ≥1.4 g of protein per kilogram of bodyweight per day [26]; and the final study also prescribed a 25% energy restriction, with a goal of 1.8 g of protein per kilogram of body weight per day, but no restrictions on times of consumption [27].

The eighth study included used a modified ADF protocol, with participants consuming ~25% of their energy requirements on every other day with ad libitum feeding on non-fasting days [28]. Participants were instructed to consume all their meals between 12 p.m. to 2 p.m. on fasting days. The comparison groups maintained their usual dietary habits.

### 3.4. Intervention—Resistance Training Protocol

The majority of studies [24,25,26,27,28,29,30] utilised a body building style workout, with 3–4 sessions per week, upper and lower or full body routines, varying from 3–15 repetitions per set and some working to failure [24,25]. Most of these studies performed the resistance training sessions in a fed state [24,25,26,27,28], with one study instructing participants to undertake their training session during their Ramadan fasting period [30], whereas a similar study compared training sessions performed during Ramadan fasting period and during the break in fasting (fed state) [29]. One study used a combination of resistance and aerobic exercise [28]. Participants completed 40 min of resistance training three times per week, varying between 10 to 15 repetitions utilising a combination of upper and lower body exercises. This was followed by at least 20 min of moderate intensity aerobic exercise (60–85% of maximal heart rate) on a motorised treadmill. Finally, in one study participants undertook two to five sessions per week at a ‘weight training gymnasium’, no other details were provided [31].

### 3.5. Effects of TRF on LBM, Body Weight and Fat

Moro and colleagues [24] reported no significant changes in LBM over the 8 weeks following TRF with an 8 h feeding window or control diet. However, the TRF group significantly reduced their fat mass (−1.6 kg ± 1.53, *p* = 0.0005) compared to the control diet. Similarly, Tinsley et al. [26] utilised an 8 h window for participants to consume their meals, but aimed for an overall small energy deficit (~250 kcal) in all groups. Despite a prescribed overall calorie deficit for both TRF and control diets, all groups combined significantly increased their body weight and LBM, with no difference between groups. Only the TRF plus HMB group experienced a significant reduction in body fat and body fat percentage compared to baseline, and only in the per protocol analysis, with intention to treat analysis showing no significant difference. While the TRF group did experience a mean reduction in these measures, this was not significant at week 8. However, all groups combined did experience a reduction in body fat percentage from baseline.

In a previous study from the same author, Tinsley et al. [25] used a shorter TRF protocol of 4 h and reported no significant changes in body composition in either the TRF or habitual diet (control) group. It should be noted that the authors did report a non-significant increase in average LBM (2.3 kg) in the habitual diet group, albeit with a small effect size (Cohen’s *d* = 0.25). More recently, Stratton et al. [27] investigated the effects of TRF with an 8 h feeding window compared to a control diet, with participants in both groups prescribed a 25% energy deficit and 1.8 g of protein per kilogram of bodyweight per day. Both groups lost significant amounts of body weight, fat mass and body fat percentage, but experienced no change in LBM, with no differences between groups.

### 3.6. Effect of Modified ADF on LBM, Body Weight and Fat

In the one study that investigated modified ADF [28], the authors separated participants into four groups: modified alternate day fasting plus exercise group, modified alternate day fasting only, exercise plus habitual diet and habitual diet only. Intention to treat analysis revealed a significant decrease in LBM (−0.4 ± 0.5 kg) in the modified ADF plus exercise group from baseline (*p* < 0.01), though this was not statistically different from the habitual diet only group. All other groups reported no significant change in LBM compared to baseline. Both modified ADF with exercise and modified ADF alone reported significant decreases in body weight (−3.3 ± 2.4 kg and −2.4 ± 3.1 kg respectively) compared to baseline, though this was only significant compared to the control diet in the combination group. The combined group also reported significant decreases in both fat mass and body fat percentage, −2.7 ± 2.0 kg and −2.5 ± 2.2%, respectively, compared to baseline, and this was significantly different to the habitual diet only group. However, the modified ADF only group reported a significant decrease in body fat percentage only (−1.26 ± 2.4%) compared to baseline.

### 3.7. Effect of Ramadan Fasting on LBM, Body Weight and Fat

Trabelsi and colleagues published two studies investigating Ramadan fasting in conjunction with resistance training four times per week in recreational bodybuilders [29,30]. In both studies, the fasting groups demonstrated no significant change in LBM, body weight, body fat mass, and body fat percentage [29,30]. In the 2012 study [30], Trabelsi incorporated a non-fasting habitual diet control group. Compared to the Ramadan fasters, the control group demonstrated a significant increase in body weight (~1.9 kg, *p* < 0.05), with non-significant increases in both fat mass and LBM. In the 2013 study [29], a strength of the study design was that authors controlled for diet prior to exercise, in order to determine if exercising in a ‘fasted’ versus ‘non-fasted, fed’ state influenced changes in body composition. Similar changes in body composition were observed for both the fasted and fed exercise groups.

The study by Stannard et al. [31] found that recreationally active males undertaking two to five sessions of ‘weight-training gymnasium’ exercise, in conjunction with Ramadan fasting, resulted in no significant changes in LBM, despite an average body weight loss of −1.3 kg (*p* = 0.019) and a −0.7 kg reduction in fat mass (*p* = 0.033).

### 3.8. Methods of Anthropometrical Assessment

The anthropometric assessment used in the studies varied with skinfold tests [29,30], bioelectrical impedance analysis (BIA) [28], dual X-ray absorptiometry (DXA) [24,25], underwater weighing [31], and a four compartment model, combining DXA and bioimpedance spectroscopy [26] or DXA, bioimpedance spectroscopy and air displacement plethysmography [27] used.

### 3.9. Reported Adherence to Dietary Protocols

Only two of the included studies reported adherence rates to the fasting protocol [25,26]. Adherence rates were calculated by dividing the number of days adherent by the total number of fasting days [25,26]. Tinsley et al. [25] reported a high level of adherence, with participants completing an average of 95.9 ± 4.1% of the 32 TRF fasting days over the 8-week duration. However, 3 of the 14 participants (21%) from the TRF intervention group were excluded from the overall analysis due to a <80% compliance, while another participant dropped out due to unknown reasons. Within the control group (habitual diet), 6 of the 14 participants (43%) did not finish the intervention. Participants also rated the difficulty of adhering to the TRF intervention using a 10 cm visual analogue scale, with a rating of zero on the left hand side of the scale indicating program adherence was ‘extremely easy’, and 10 on the right hand side indicating adherence was ‘extremely difficult’. After 4 weeks, difficulty was rated at 3.6 ± 1.4 out of 10, and did not change significantly by the end of the study (3.8 ± 2.2, *p* = 0.86). Some participants reported difficulty initially with the fast regime, but commented that it became easier after several days. In their other study, Tinsley et al. [26] reported a 89 ± 8% compliance with their meal timing protocol, based on intention to treat analysis, increasing to 91 ± 3% using per protocol analysis. Participants were excluded from the per protocol analysis if they had less than 80% adherence to the eating schedule, or completed less than 22 of 24 resistance training sessions. Overall, 12 of 13 participants allocated to the TRF and 10 of 13 in the TRF plus HMB group completed the study, while only 9 of 14 completed in the control group. In other studies, adherence was monitored by participants completing dietary logs every day during the intervention period [28] or through a structured interview performed by a dietitian [24]. However, dietary data and adherence was not reported. None of the Ramadan fasting studies included measured adherence to the protocol.

### 3.10. Quality Appraisal

All studies included in the present review scored between 16–20 points on the Downs and Black checklist out of a possible 27 [24,25,26,27,28,29,30,31].

## 4. Discussion

To the authors’ knowledge, this is the first review to systematically analyse the published literature on the combined effects of IF and resistance training on changes in LBM in recreationally active and non-elite trained individuals. The major finding is that, despite heterogeneity in study design, especially fasting method, LBM was generally maintained when IF and resistance training were undertaken concurrently during the short to medium term. Though four studies observed mean increases in LBM [24,26,29,31], only one study reported a statistically significant increase (0.9–1.2 kg) [26]. Interestingly, the study that reported the statistically significant increase in LBM also observed a mean increase in participants’ body weights, which is in contrast to all other studies, and indicates that energy balance may be a potential contributing factor. Thus, it remains unclear whether LBM accrual is possible when fasting, combined with resistance training, results in an overall energy deficit. Additionally, the length of the intervention may also be a factor given most studies were only 4–8 weeks in duration. Therefore, further research is needed to examine the longer term effects of these interventions, but also other forms of IF, with the majority of studies focusing on TRF and Ramadan style fasting.

For many individuals, the primary goal of resistance training is to increase LBM, or at the very least, maintain it. Of the eight studies included and reviewed, only one study showed a reduction in LBM from baseline when IF and resistance training were undertaken together [28]. However, it should be noted that this reduction was small, and although significant, was not significantly different to the other experimental groups in the study [28]. This small reduction could be due to the addition of aerobic exercise (20 min) at the end of each resistance training session with evidence suggesting that concurrent training (performing both resistance and aerobic exercise together) may compromise resistance training-induced muscle adaptations due to the “concurrent training effect” (CTE) [32]. However, whether CTE is the reason for the LBM reduction in the Oh et al. [28] study is unknown.

Whilst the majority of studies included suggest that LBM can be maintained when undertaking IF and resistance training together, it is less clear whether or not the accrual of LBM is compromised. A number of studies did report mean increases in LBM [24,26,29,31], however, only one study reported a statistically significant increase (0.9–1.2 kg) [26]. This was evident by a main effect for time, with no difference (interaction) between the TRF or normal diet groups. Of particular note is that, despite participants in this study being prescribed an energy deficit of ~250 kcal per day, energy intake actually increased over the intervention period, which led to an increase in mean weight for all groups by the end of the study. Given this was the only study to show a meaningful and significant increase in LBM, it does suggest that an apparent energy surplus (as evidenced by a mean increase in weight) could be a major determinant in supporting training-induced adaptations, such as muscle growth [33]. Therefore, it raises an important question of whether IF compromises growth of LBM when undertaking resistance training without an apparent energy surplus. Interestingly, changes in LBM were not statistically different between intervention and ‘normal diet’ control groups in the studies that included such a comparative group [24,25,26,27,28,30]. This is somewhat surprising, given most, though not all, resistance training interventions lead to an increase in LBM [34]. It is possible the length of training (i.e., 4–8 weeks) used in the reviewed studies did not allow adequate time for significant growth to occur. Although the time-course of muscle hypertrophy is poorly defined, it is usually expected to begin within a couple of months for untrained individuals, with those with prior training experience observing the effects later [35]. Furthermore, while most training programs were of similar frequency across studies (i.e., 3–4 sessions per week), the number of repetitions, load and whether or not participants worked to failure varied, which may have affected total volume of work. Indeed, variation in overall volume, as opposed to frequency, seems to have greater impact on muscle growth [36]. Thus, it is likely that the relatively short durations and variations in volume/effort of training programs used in the reviewed studies, rather than IF per se was an important determining factor in the observed effects on LBM. It is clear that further research is needed that incorporates longer training programs (>8 weeks), to gain a better understanding of the impact of IF on training-induced adaptations.

Protein intake is another major determinant of training-induced changes in LBM [37]. Of the six studies that reported it [24,25,26,27,29,30], protein intake ranged from 1.2 g/kg/day to 1.9 g/kg/day, which is close to or above the level recommended for LBM maintenance and accrual [37]. In the one study that reported a LBM loss [28], no protein intake was reported, and indeed no emphasis appeared to be given to protein intake for participants. While it is possible that insufficient protein intake could be a contributing factor for the observed LBM loss, participants in this study also lost the most weight compared to all other included studies, possibly due to a combination of the more severe energy restriction protocol used and the extra aerobic exercise component included. Thus, larger weight reductions and periods of greater energy deficit (though not directly measured) could also be a contributing factor.

The observed effects of IF and resistance training on fat mass and body fat percentage were less consistent. Five studies reported statistically significant changes in body fat mass or body fat percentage [24,26,27,28,31]. The remaining three studies [22,26,27] reported no significant changes. Body fat losses ranged from an average of 0.7 kg to 2.7 kg, while body fat percentage reductions ranged from 0.8–2.5%. The variability in fat loss is likely a reflection of intervention duration, total energy intake and participant characteristics, such as sex and baseline weight status. The modified ADF study [28] demonstrated the greatest weight and fat loss compared to other fasting protocols. This is to be expected given they prescribed the most severe energy restriction, included an extra aerobic exercise component and had the equal longest duration. However, weight loss does not appear to be a pre-requisite for changes in body fat or body fat percentage in the studies reviewed. While four of the five studies that showed changes in these measures resulted in (generally small) concomitant weight loss [24,27,28,31], one study showed a reduction in fat, despite a gain in weight [26]. This provides evidence that IF with resistance training can improve body composition (at least with regard to body fat loss) independent of large energy deficits. Changes in metabolic fuel preference during fasting is a likely mechanism behind the enhanced fat loss, with a decline in carbohydrate oxidation and increases in fat oxidation, ketogenesis and gluconeogenesis evident [38,39]. However, whether these contribute to meaningful changes in body fat levels requires further investigation.

There are several limitations associated with this review and the included studies. Firstly, our review only considered changes in whole body LBM, which does not capture local changes in muscle hypertrophy. Half of the included studies (all TRF studies) did use more sensitive assessments of muscle hypertrophy (i.e., muscle ultrasound). While two studies showed similar directional change in comparison to the whole body LBM changes assessed by DXA or a 4C model [24,27], the other two reported conflicting results [26,27]. Given the majority of studies comparing these different techniques have been undertaken in clinical (i.e., sarcopenic) populations, it is clear that further work is needed in populations included in this review. Secondly, most studies did not include an IF only comparator group, making it unclear whether there is an additive effect of resistance training on LBM compared to IF alone. Thirdly, the included studies were all of a short to medium duration, and results cannot be generalised to longer intervention periods. Finally, while the authors tried make our search comprehensive, we focused on the published literature only.

## 5. Conclusions

Lean body mass is generally maintained when IF, including when followed for religious reasons, is combined with resistance training. However, whether or not IF inhibits LBM accrual is unclear, and may be contingent on the adequate provision of protein, and energy balance. The combination of IF and resistance training may also lead to a reduction in body fat, not only during apparent energy deficit, but also energy surplus. Given low muscle mass and poor muscle strength are important risk factors for disability and potentially mortality, especially in older individuals, these findings may also have important clinical implications. Future research should aim to examine the longer-term effects of various IF regimes with resistance training on LBM, incorporating varying levels of energy intake, and, where possible, appropriate non-exercise and non-fasting control groups. Furthermore, given the growing popularity of IF for weight loss, future research should also consider whether IF paired with resistance training is more or less effective and sustainable compared to other traditional forms of energy restriction diets.

## Figures and Tables

**Table 1:** Intermittent fasting combined with exercise and effect on body composition.

Author, Year	Participant Baseline Characteristics	Drop Outs (Final Number of Completers)	Duration of Study	Description of Fasting	Description of Exercise	Weight Change	Body Composition Changes	Method of Anthropometric Assessment	Quality Score (Downs and Black)	Fasted During Exercise?
**Time Restricted Feeding Studies**										
Moro et al. (2016) [24]	*n* = 34 Male Resistance trained (at least 3–5 times/week for 5 years) **Time restricted feeding group** *n* = 17 29.9 ± 4.1 years old BMI 26.5 kg/m^2^ * BF% 13.0% * Control diet group *n* = 17 28.5 ± 3.5 years old BMI 27.2 kg/m^2^ * BF% 13.2% *	NR—assumed full completion	8 weeks	**Time restricted feeding** 100% of energy needs consumed over 3 meals in an 8 h window (1 p.m., 4 p.m. and 8 p.m.), 20 g of whey protein after training **Control** 100% of energy needs consumed over 3 meals across the day (8 a.m., 1 p.m. and 8 p.m.), 20 g of whey protein after training	**Both groups** 3 resistance sessions/week, split routine, 6–8 repetitions at 85–90% of 1 RM to failure, supervised sessions, conducted between 4–6 p.m.	**Time restricted feeding** ↓ 1.0 kg **Control** ↑ 0.2 kg	**Time restricted feeding** LBM ↑ 0.6 kg (NS) FM ↓ 1.6 kg **Control** LBM ↑ 0.5 kg (NS) FM ↓ 0.3 kg (NS)	DXA	18	No
Tinsley et al. (2017) [25]	*n* = 28 Male Both groups recreationally active; have not followed a consistent RT programme over previous 3 months **Time restricted feeding group** *n* = 14 22.9 ± 4.0 years old BMI 27.2 kg/m^2^ * BF% 21.3 ± 5.4% **Normal diet group** *n* = 14 22.0 ± 2.4 years old BMI 24.3 kg/m^2^ * BF% 18.7 ± 3.8%	**Time restricted feeding group** 1 (13 completers) **Normal diet group** 5 (9 completers)	8 weeks	**Time restricted feeding** 4 days/week (non-training days) all energy consumed in a 4 h window between 4 p.m. and midnight 3 days/week ad libitum **Normal diet** Usual dietary patterns	**Both groups** 3 resistance sessions/week on non-consecutive days, alternating upper and lower body, 8–12 reps to failure, 4 sets of each exercise	**Time restricted feeding** ↓ 1.0 kg (NS) **Normal diet** ↑ 3.0 kg (NS) Analysis based on *n* = 10 for time restricted feeders and *n* = 8 normal diet due to exclusion for low compliance	**Time restricted feeding** LBM ↓ 0.2 kg (NS) FM ↓ 0.6 kg (NS) **Normal diet** LBM ↑ 2.3 kg (NS) FM ↑ 0.8 kg (NS) Analysis based on *n* = 10 for time restricted feeders and *n* = 8 normal diet due to exclusion for low compliance	DXA	16	No
Tinsley et al. (2019) [26]	*n* = 40 Female Resistance trained (2 to 4 sessions per week for at least 1 year); BF% less than 33% **Time restricted feeding group** *n* = 13 23.3 ± 1.5 years old BMI 23.8 kg/m^2^ * BF% 28.4 ± 1.5% **Time restricted feeding group plus HMB** *n* = 13 22.3 ± 3.4 years old BMI 22.9 kg/m^2^ * BF% 28.7 ± 1.5% **Control diet group** *n* = 14 22.6 ± 2.7 years old BMI 22.5 kg/m^2^ * BF% 29.3 ± 1.5%	**Time restricted feeding group** 1 (12 completers) Time restricted **feeding group plus HMB** 3 (10 completers) **Control diet group** 5 (9 completers)	8 weeks	**Time restricted feeding** All energy consumed between 12 p.m. and 8 p.m. each day, prescribed energy deficit of 250 kcal and protein of ≥1.4 g/kg/d. Instructed to consume whey protein supplement each day to achieve protein target **Time restricted feeding plus HMB** Same as time restricted feeding group, plus 3 g/day HMB **Control diet group** Energy and protein targets as per the time restricted feeding group, however instructed to consume breakfast upon waking, and continue eating at self-selected intervals	**All groups** 3 resistance sessions/week on non-consecutive days, alternating 2 different upper and lower body sessions Conducted between 12 p.m. and 6 p.m.	**Time restricted feeding group** ↑ 0.6 kg **Time restricted feeding group plus HMB** ↑ 0.6 kg **Control diet group** ↑ 1.1 kg Data from ITT analysis. Results significant for all groups combined	**Time restricted feeding group** FM ↓ 0.4 kg LBM ↑ 0.9 kg BF% ↓ 0.8% **Time restricted feeding group plus HMB** FM ↓ 0.7 kg LBM ↑ 1.2 kg BF% ↓ 1.4% **Control diet group** FM ↑ 0.4 kg LBM ↑ 0.9 kg BF% ↑ 0.1% Data from ITT analysis. Results significant for all groups combined	4C	20	No
Stratton et al. (2020) [27]	*n* = 32 Male Recreationally trained (2–4 sessions per week for at least 6 months) **Time restricted feeding group** *n* = 13 22.9 ± 3.6 years old BMI 25.9 kg/m^2^ * BF% 19.9 ± 8.3% **Control diet group** *n* = 13 22.5 ± 2.2 years old BMI 26.4 kg/m^2^ * BF% 18.9 ± 7.4%	**Time restricted feeding group** 0 (16 completers **Control diet group** 2 (14 completers)	4 weeks	**Time restricted feeding** All energy consumed between 12 p.m. and 8 p.m., or 1 m and 9 p.m. each day, prescribed 25% energy deficit and protein intake of 1.8 g/kg/d. Provided 50 g whey protein supplement on training days. **Control diet group** Energy and protein targets as per the time restricted feeding group, but with no time restrictions on consumption. Additionally provided with 50 g whey protein supplement on training days	**Both Groups** 3 full body resistance sessions/week. Conducted between 3 p.m. and 8 p.m.	**Time restricted feeding** ↓ 1.2 kg **Control diet** group ↓ 1.4 kg	**Time restricted feeding** FM ↓ 1.5 kg LBM NS change (actual values NR) BF% ↓ 1.6% **Control diet group** FM ↓ 1.4 kg LBM NS change (actual values NR) BF% ↓ 1.5% Analysis based on *n* = 13 for time restricted feeders and *n* = 13 for control diet group due to exclusion for low compliance	4C	19	No
**Modified Alternate Day Fasting Study**										
Oh et al. (2018) [28]	*n* = 45 Training history unclear, but described as ‘unfamiliar with resistance exercise’ **Alternate day fasting + exercise group** *n* = 12 male = 5, female = 7 37.3 ± 7.3 years old BMI 27.5 ± 2.6 kg/m^2^ BF% 34.2 ± 6.1% **Alternate day fasting group** *n* = 13 male = 3, female = 10 32.9 ± 7.3 years old BMI 27.6 ± 2.8 kg/m^2^ BF% 34.9 ± 4.6% **Exercise only group** *n* = 10 male = 7, female = 3 35.7 ± 7.9 years old BMI 28.3 ± 4.1 kg/m^2^ BF% 31.0 ± 5.0% **Normal diet group** *n* = 10 male = 4, female = 6 40.6 ± 10.0 years old BMI 26.3 ± 3.0 kg/m^2^ BF% 32.2 ± 4.4%	**Alternate day fasting + exercise group** 2 (10 completers) **Alternate day fasting group** 4 (9 completers) **Exercise only group** 2 (8 completers) **Normal diet group** 2 (8 completers) Sex of drop outs NR	8 weeks	**Alternate day fasting groups** 75% Calorie restriction alternating with ad libitum feeding **Normal diet/exercise only groups** No instruction given	**Alternate day fasting + exercise and normal diet + exercise groups** 3 training sessions per week consisting of 40 min of resistance training followed by 20 min of aerobic exercise on a treadmill. Resistance training was upper and lower body, 3 different sessions each week. Intensity ranged from 70% 10 RM (15 repetitions) to 100% 10 RM (10 repetitions) and altered each week. Aerobic exercise performed at 60–85% age predicted maximal heart rate	**Alternate day fasting + exercise group** ↓ 3.3 ± 2.4 kg **Alternate day fasting group** ↓ 2.4 ± 3.1 kg **Exercise only group** NS change **Normal diet group** NS change Data from ITT analysis	**Alternate day fasting + exercise** FM ↓ 2.7 ± 2.0 kg LBM ↓ 0.4 ± 0.5 kg BF% ↓ 2.5 ± 2.2% **Alternate day fasting group** FM ↓ 1.6 ± 2.3 kg (NS) LBM ↓ 0.5 ± 0.9 kg (NS) BF% ↓ 1.3 ± 2.4% **Exercise only group** FM ↓ 1.2 ± 1.9 kg LBM ↓ 0.1 ± 0.9 kg (NS) BF% ↓ 1.1 ± 1.8% (NS) **Normal diet group** FM ↓ 0.3 ± 1.3 kg (NS) LBM ↓ 0.2 ± 0.7 kg (NS) BF% ↓ 0.1 ± 1.5% (NS) Data from ITT analysis	BIA	18	No
**Ramadan Fasting Studies**										
Trabelsi et al. (2013) [29]	*n* = 16 Male Resistance trained (3 times/week for 1.6 ± 0.6 and 1.5 ± 0.5 years) **Fasted exercise *n* = 8** 25.0 ± 3.0 years old BMI 25.8 ± 4.0 kg/m^2^ BF% 15.0 ± 2.0% **Fed exercise *n* = 8** 25.0 ± 2.0 years old BMI 26.0 ± 1.7 kg/m^2^ BF% 14.0 ± 1.0%	NR	4 weeks	**Ramadan fasting** Average fast 15 h	**Both groups** 4 resistance sessions/week, 4–6 exercises, 4 sets at 10 RM, split routine, supervised **Fasted exercise group** Exercise conducted between 4–6 p.m. before breaking fast **Fed exercise group** Exercise conducted between 9–10 p.m.	NS change in either group	**Fasted exercise group** LBM ↓ 0.2 kg (NS) BF% ↓ 0.7% (NS) **Fed Exercise group** LBM ↑ 0.3 kg (NS) BF% ↓ 0.4% (NS)	Skinfolds	17	Mixed—exercise conducted between 4–6 p.m. for fasted group, unclear when fast began
Trabelsi et al. (2012) [30]	*n* = 16 Male Recreational bodybuilders (at least 1 year experience) **Fasters *n* = 9 (it is unclear whether this data is duplicated from Trabelsi et al. 2013)** 24.0 ± 3.0 years old BMI 26.0 ± 0.7 kg/m^2^ BF% 14.5 ± 2.0% **Non fasters *n* = 7** 26.0 ± 3.0 years old BMI 26.0 ± 1.5 kg/m^2^ BF% 13.5 ± 1.4%	NR	4 weeks	**Ramadan fasting** Average fast 15 h **Control group** Normal diet	**Both groups** 4 resistance sessions/week, 4–6 exercises, 4 sets at 10 RM, split routine, supervised	**Fasters** NS change **Non Fasters** ↑ 1.9 kg	**Fasters** FM ↓ 0.6 kg (NS) LBM ↓ 0.1 kg (NS) BF% ↓ 0.7% (NS) **Non Fasters** FM ↑ 1.2 kg (NS) LBM ↑ 0.7 kg (NS) BF% ↑ 1.1% (NS)	Skinfolds	16	Yes—exercise conducted between 4–6 p.m., unclear when fast began
Stannard et al. (2008) [31]	*n* = 8 Male Recreationally active (2–5 sessions/week) 24.1 ± 0.8 years old BMI 24 kg/m^2^ * BF% 12.9 ± 3.5%	NR	4 weeks	**Ramadan fasting** Average fast 14.5 h	2–5 sessions/week in the ‘weight-training gymnasium’. Type and duration of exercise is unspecified	↓ 1.3 kg	FM ↓ 0.7 kg LBM ↑ 0.1 kg (NS) BF% ↓ 0.7% (NS)	Underwater weighing	16	Unclear

Notes: All results presented as mean ± SD. Where no SD has been reported in the original study, values are means. Baseline characteristics are representative of all participants before drop outs. * Data not reported, calculated by the authors from baseline height and weight (BMI), or from baseline weight and fat mass for BF%. 1 RM = 1 repetition maximum, 10 RM = 10 repetition maximum, 4C = 4 compartment model, BF% = body fat percentage, BMI = body mass index, DXA = dual X-ray absorptiometry, FM = fat mass, HMB = β-hydroxy β-methylbutyrate, ITT = intention to treat, LBM = lean body mass, NS = non-significant change, NR = not reported, PP = per protocol, RT = resistance training.

## Supplementary Materials

The following are available online at https://www.mdpi.com/2072-6643/12/8/2349/s1, Table S1: PRISMA checklist, Table S2: Full search strategy example.
