# Supplementary material for: The Effects of Intermittent Fasting Combined with Resistance Training on Lean Body Mass: A Systematic Review of Human Studies
Source: Nutrients. 2020 Aug 6;12(8):2349. doi: 10.3390/nu12082349 (PMC7468742; doi:10.3390/nu12082349)
Supplement: Supplementary file 1 [file nutrients-12-02349-s001.zip › Supplementary material 2 full search strategy.docx]

Supplementary Material 2. Full Electronic Search Strategy for MEDLINE

| 1 | (alternate day fast* or alternat* calori* diet* or alternate day diet* or alternate day modified fast* or intermittent fast* or intermittent energy fast* or intermittent energy restrict* or intermittent calori* restrict* or ADF or time restricted feed* or TRF or ramadan or ramadan fast*).mp. [mp=title, abstract, original title, name of substance word, subject heading word, floating sub-heading word, keyword heading word, protocol supplementary concept word, rare disease supplementary concept word, unique identifier, synonyms] | 6660 |
| --- | --- | --- |
| 2 | (exercis* or resistance exercis* or training).mp. [mp=title, abstract, original title, name of substance word, subject heading word, floating sub-heading word, keyword heading word, protocol supplementary concept word, rare disease supplementary concept word, unique identifier, synonyms] | 740194 |
| 3 | body composition.mp. or exp Body Composition/ | 57128 |
| 4 | 1 and 2 and 3 | 46 |
